# Correction: Cuenca-Zamora, Ernesto José., et al. Tubulin in Platelets: When the Shape Matter. *Int. J. Mol. Sci.* 2019, *20*, 3484

**DOI:** 10.3390/ijms21103577

**Published:** 2020-05-19

**Authors:** Ernesto José Cuenca-Zamora, Francisca Ferrer-Marín, José Rivera, Raúl Teruel-Montoya

**Affiliations:** 1 Servicio de Hematología y Oncología Médica, Centro Regional de Hemodonación, Hospital Universitario Morales Meseguer, IMIB-Arrixaca, Red CIBERER CB15/00055, 30003 Murcia, Spain; ernestojose.cuenca@um.es (E.J.C.-Z.); jose.rivera@carm.es (J.R.); raulteruelmontoya@hotmail.com (R.T.-M.); 2 Grado de Medicina, Universidad Católica San Antonio (UCAM), Campus de los Jerónimos, 30107 Murcia, Spain

The authors wish to make the following corrections to this paper [1]:

To change minimally the Acknowledgments section as follows below, to more appropriately recognize support and funding:

Current

Acknowledgments: The authors thank Eva Caparrós-Pérez and Nataliya Bohdan for their technical support. This work was supported by a research grant from Instituto de Salud Carlos III (ISCIII) (PI18/00316 and PI17/01311) and Séneca 20644/JLI/18. Research by the authors’ group is further supported by grants from CIBERER (CB15/00055) Grupo CIBERER 765 and Fundación Séneca 19873/GERM/15.

Corrected

Acknowledgments: The authors thank Eva Caparrós-Pérez and Nataliya Bohdan for their technical support. This work was supported by research grants from Instituto de Salud Carlos III and FEDER (ISCIII & Feder) (PI18/00316 and PI17/01311) and Séneca 20644/JLI/18. Research by the authors’ group is further supported by CIBERER (CB15/00055) and Fundación Séneca 19873/GERM/15.

The authors would like to apologize for any inconvenience caused to the readers by these changes.
